# Silymarin Modulates Microbiota in the Gut to Improve the Health of Sow from Late Gestation to Lactation

**DOI:** 10.3390/ani12172202

**Published:** 2022-08-26

**Authors:** Shengyu Xu, Xiaojun Jiang, Xinlin Jia, Xuemei Jiang, Lianqiang Che, Yan Lin, Yong Zhuo, Bin Feng, Zhengfeng Fang, Jian Li, Jianping Wang, Zhihua Ren, De Wu

**Affiliations:** 1 Key Laboratory of Animal Disease-Resistance Nutrition, Ministry of Education, Animal Nutrition Institute, Sichuan Agricultural University, Chengdu 611130, China; 2 Key Laboratory of Animal Disease-Resistance Nutrition and Feed, Ministry of Agriculture and Rural Affairs, Animal Nutrition Institute, Sichuan Agricultural University, Chengdu 611130, China; 3 College of Veterinary Medicine, Sichuan Agricultural University, Chengdu 611130, China

**Keywords:** inflammation, microbiota, silymarin, sow

## Abstract

**Simple Summary:**

The gut microbiome is crucial for lactation sow health and production. This study aimed to investigate the effect of dietary silymarin supplementation on the inflammatory responses and fecal microbiota in lactation sow and to evaluate the relationship between the inflammatory factor and fecal microbiota in lactation sow. It is suggested in our results that silymarin supplementation decreased inflammatory responses of lactation sow and changed fecal microbiota composition at different taxonomic levels. The altered fecal microbiota was associated with variations in inflammatory factors, indicating that silymarin modulates microbiota in the gut and may improve the health of lactation sow.

**Abstract:**

Inflammatory responses reduce milk production in lactating sows. Silymarin may modulate inflammatory reactions. Here, we aimed to verify whether dietary silymarin supplementation could alleviate inflammatory responses in lactating sows through microbiota change in the gut. We also investigated how silymarin impacts inflammatory response in lactating sows. One hundred and ten sows were randomly assigned to a control diet (basal diet) or treatment diet (basal diet and 40 g/d silymarin) from the 108th day of gestation to weaning. Blood, milk, and feces from sows were collected for analysis. It was shown in the results that dietary silymarin supplementation decreased the level of pro-inflammatory cytokine IL-1β (*p* < 0.05) on the 18th day of lactation in the blood of the sows. Dietary silymarin supplementation tended to decrease (*p* = 0.06) somatic cell count in the colostrum of sows. Dietary silymarin supplementation reduced the gut bacterial community and the richness of the gut microbial community (*p* < 0.01) using 16S rRNA gene sequencing. The fecal microbes varied at different taxonomic levels in the lactating sows with silymarin supplementation. The most representative changes included an increase in the relative abundance of Fibrobacteres and Actinobacteria (*p* < 0.05) and tended to reduce the relative abundance of Spirochaetaes and Tenericutes (*p* = 0.09, 0.06) at the phylum level. It is suggested that dietary silymarin supplementation in late gestation until lactation has anti-inflammatory effects in lactation sow, which could be associated with the modulation of gut microbiota.

## 1. Introduction

Silymarin is a 25-carbon flavonolignan mixture extracted from the plant *Silybum marianum* (L.) Gaertn. (Asteraceae) (milk thistle) [1]. The main components of silymarin are four isomers: silybin, isosilybin, silydianin, and silychristin [2]. In our previous study, dietary silymarin supplementation during the sow transition and lactation reduced oxidative stress, decreased TNF-α concentration in serum on the seventh day of lactation, and increased the colostrum yield of sows [3]. Sows fed micelle silymarin from the 109th prenatal day to the 21st postnatal day increased their feed intake and milk yields during lactation, along with increased sow antioxidant capacity [4]. It has been found in previous studies that the functional mechanism of silymarin includes anti-inflammatory, anti-oxidation, scavenging free radicals, regulating intracellular glutathione peroxidase content, stabilizing cell membrane, regulating cell permeability, and improving liver regeneration ability [5,6,7]. However, there remains little knowledge of the maternal diversity of gut microbiota following silymarin supplementation during late gestation to lactation. The flavonoids extracted from *S. marianum* were active against *Staphylococcus aureus*, *Staphylococcus albus*, *Candida albicans*, and *Saccharomyces cerevisiae* [8]. This suggests that dietary silymarin supplementation leads to variation in the microbiota in the gut of sows.

Placental mitochondria and mitochondrial electron chain enzyme activity increase in sows, especially in those before farrowing, with the rise of gestational age and fetal metabolic intensity. This process increases reactive oxygen species (ROS) and maternal oxidative stress [9]. Cell-generated ROS can activate the tumor necrosis factor-α (NF-κB) signaling pathway [10], leading to an inflammation response that adversely affects sow reproductive performance and health [11,12]. Furthermore, gut microbiota plays a critical role in the metabolism of nutrients, immune function, and the health of animals. At the same time, the microbiota is also regulated by the nutrient intake and immune and metabolic status of the host. The transition period from gestation to lactation is a crucial window for microbiota remodeling in sows [13]. The top dominant phyla were Firmicutes (35.67–73.96%), Bacteroidetes (11.95–51.43%), Proteobacteria (3.40–6.54%), Tenericutes (2.50–5.69%), and Spirochaetes (0.98–9.87%) on the 14th and the 21st day of lactation of sows [13,14]. However, whether silymarin supplementation in the diet of sows from late gestation to lactation has a correlation between sow gut microbiota and inflammation and then affects the health of the sow remains to be further investigated. Therefore, this study aimed to evaluate the hypothesis that silymarin supplementation might modulate microbiota in the gut of sows and change the inflammatory response, thus affecting the health of lactation sows.

## 2. Materials and Methods

The experimental procedures were approved by the Guide for the Care and Use of Laboratory Animals prepared by the Animal Care and Use Committee of Sichuan Agricultural University. Silymarin was provided by Tianben Bio-Engineering Co., Ltd. (Xi’an, China), which contained 10.32% silybin, 15.64% silydianin plus silychristin, and 6.91% isosilybin as the main ingredients.

### 2.1. Animals and Experimental Design

A total of 110 sows (Landrace × Yorkshire, parity 1–8, 19.50 ± 0.36 mm backfat thickness) were used. Half of the sows in each parity were fed a control diet (CTL) (basic diet, *n* = 55) or silymarin diet (TRT) (basic diet + 40 g/d silymarin, *n* = 55) from the 108th day of gestation until weaning. The basal diet for this study was the same as in our previous study [3]. The basal lactation diet contained 14.22 MJ of digestible energy per kilogram (DE/kg), 17.00% crude protein, 1.06% Lys, 0.80% calcium, and 0.35% standardized total tract digestible phosphorus.

Sows were housed in individual farrowing crates on the 108th day of gestation. Sows were fed an average diet of 3.5 kg/d and two times/d during the late gestation stage, fed a 1.34 kg of diet on the day of farrowing, and then gradually increased by 0.5 kg/d and two times/d up to the maximum amount of feeding. During the experiment, free access to water was maintained.

### 2.2. Sample Collection

Colostrum samples were collected within one hour after the end of farrowing from multiparous sows (*n* = 10/treatment group, parity 2–4, randomly selected) and stored at -20 °C until analysis. On the 18th day of lactation, 20 mL milk samples (*n* = 10/treatment group, same sow that collected colostrum sample) were collected after injection of 1.0 mL of oxytocin (20 IU/mL, Hangzhou Animal Drug Factory, Hangzhou, China) via the ear vein. One tube of milk sample was stored at −20 °C until analysis. Another milk tube was centrifuged at 8000× *g* for 15 min at 4 °C to obtain milk serum and then stored in a −20 °C freezer for later use.

Blood samples were collected (*n* = 10/treatment group, sow, which collected milk samples) on the 18th day of lactation through ear venipuncture. After centrifuging at 3000× *g* for 15 min at 4 °C, a serum sample was obtained, kept in liquid nitrogen, and stored at –20 °C until analysis.

For each treatment, 10 sows were selected, and their colostrum was taken. In the early morning on the 18th day of lactation, three tubes of fresh feces (≈5 g/tube) were directly collected by massaging the rectum of each sow. Fresh feces were stored in a sterile tube and kept in liquid nitrogen before being transferred to −80 °C.

### 2.3. Somatic Cell and Cytokine Analyses

The somatic cell count (SCC) in milk was determined using a FOSS MATIC 5000 (Foss Electric A/S, Hillerød, Denmark). Serum and milk serum interleukin-1β (IL-1β), interleukin-6 (IL-6), interleukin-10 (IL-10), and tumor necrosis factor-α (TNF-α) concentrations were analyzed by corresponding ELISA assay kits (Nanjing Jiancheng Bioengineering Institute, Nanjing, China; porcine specific antibodies) according to the manufacturer’s instructions.

### 2.4. Bacterial Community Analysis

The composition of the microbial community in the feces (*n* = 10/treatment group) was analyzed by high-throughput pyrosequencing, as previously described [15]. The sequencing and bioinformatics analyses were performed by Novogene Bioinformatics Technology Co. (Beijing, China).

The data were analyzed following our previous protocol [13]. Briefly, high-quality tags were filtered and clustered into operational taxonomic unit (OTU) utilizing Uparse v7.0.1001 (http://drive5.com/uparse/, accessed on 20 December 2019) at 97% sequence similarity. The Ribosomal Database Project (RDP) classifier Version 2.2 (http://github.com/rdpstaff/ (accessed on 20 December 2019)) was applied to assign a taxonomy for 16S rRNA gene sequences. The representative sequence of OTUs was annotated. A Venn diagram was generated to compare the OTUs of the treatment groups. Alpha diversity values for each sample were assessed using Qiime 1.7.0. Principal coordinates analysis (PCoA) plots were produced using unweighted UniFrac metrics.

### 2.5. Statistical Analysis

Before the analyses, descriptive statistics were performed to check the normality and homogeneity of variances. The data were analyzed using t-tests in SAS 9.4 (SAS Institute, Cary, NC, USA). The results are presented as mean ± SE, except for microbial data, which are represented by mean ± SD. *p* < 0.05 was considered statistically significant, and 0.05 ≤ *p* < 0.10 was considered a tendency.

Correlations between gut microbiota and sow serum cytokines were analyzed with Spearman’s correlation in R 3.0.2 with the Rstudio 0.97.310 package. Differences of *p* < 0.05 were considered statistically significant, and 0.05 ≤ *p* < 0.10 was considered a tendency.

## 3. Results

### 3.1. Effect of Dietary Silymarin Supplementation on the Somatic Cell Count of Lactating Sows

Dietary supplementation with silymarin in late gestation and lactation tended to decrease somatic cell count in the colostrum of sows (*p* = 0.06, Table 1). Still, no effect on milk somatic cell count on the 18th day of lactation was detected in the sows.

### 3.2. Effect of Dietary Silymarin Supplementation on the Cytokines of Lactating Sows

Dietary silymarin supplementation in late gestation and lactating sows decreased IL-1β concentration in the blood on the 18th day of lactation (*p* < 0.01, Table 2). No difference in blood serum was observed in IL-6, IL-10, and TNF-α between the treatment and control groups. No difference was observed in the cytokines in the milk serum of lactating sows.

### 3.3. Effect of Silymarin on Microbiota in the Feces of Lactating Sows

**Figure 1:**
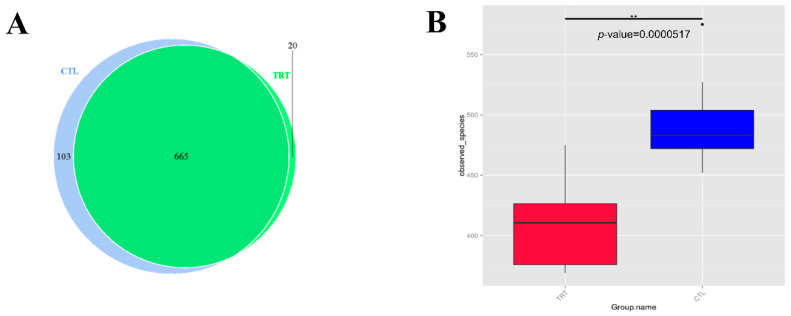
Dietary silymarin supplementation modulated microbiota diversity in lactation sow. (**A**) A Venn diagram was generated to describe the common and unique OTUs between the treatment groups. (**B**) The effect of silymarin on the observed species. CTL, sow fed the basal diet; TRT, sow fed basal diet with silymarin 40 g/d from late gestation to lactation. ** are significantly different (*p* < 0.01).

**Figure 2:**
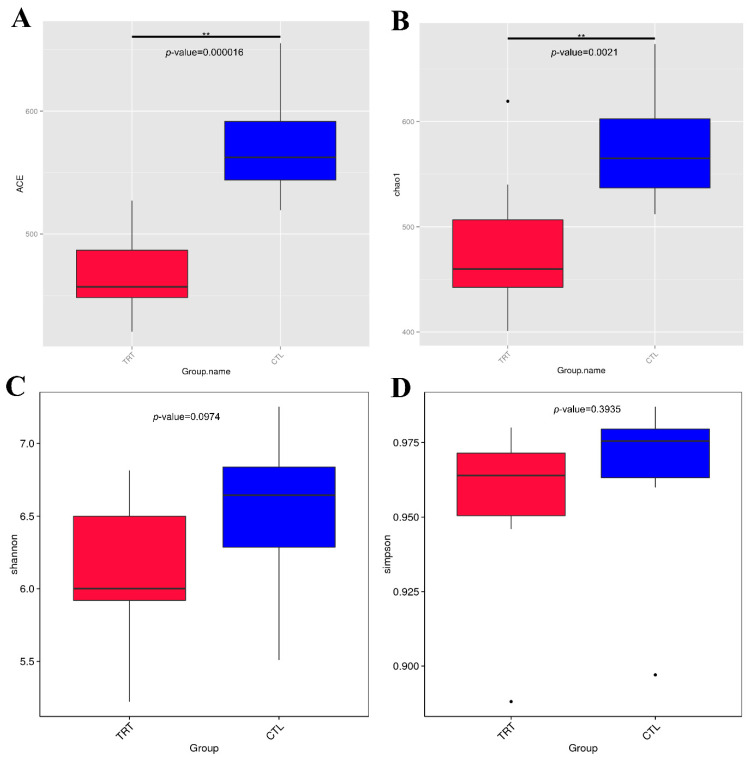
Dietary silymarin supplementation modulated the microbiota alpha diversity index of lactation sow, including richness (ACE, Chao 1 index) and diversity (Shannon, Simpson index). (**A**) ACE index: *p* < 0.01. (**B**) Chao 1 index: *p* < 0.01. (**C**) Shannon index: *p* = 0.097. (**D**) Simpson index: *p* = 0.39. *n* = 10. CTL, sow fed the basal diet; TRT, sow fed basal diet with silymarin 40 g/d from late gestation to lactation. ** are significantly different (*p* < 0.01).

**Figure 3:**
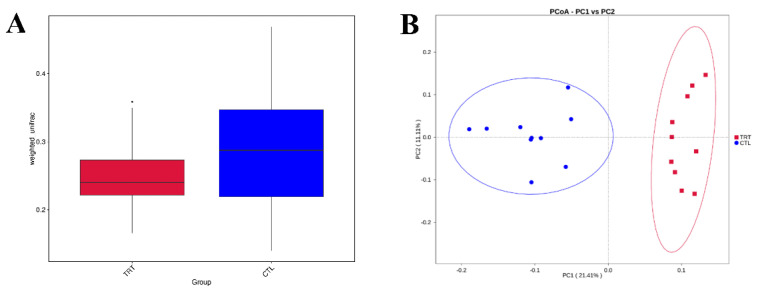
Dietary silymarin supplementation modulated microbiota beta diversity in lactation sow. (**A**) Beta diversity analysis based on Weighted Unifrac distance. (**B**) Principal coordinates analysis of each sample based on unweighted UniFrac metric. *n* = 10. CTL, sow fed the basal diet; TRT, sow fed basal diet with silymarin 40 g/d from late gestation to lactation.

A total of 20 fecal samples were subjected to 16S rRNA gene sequencing. A set of 665 OTUs existed in the two groups and was defined as core OTUs (Figure 1). Dietary supplementation with silymarin in late gestation and lactation reduced the microbial diversity in the feces of lactating sows (Figure 1A,B). Silymarin also reduced the richness (ACE, Chao1 index, *p* < 0.01, Figure 2A,B) and diversity (*p* < 0.1, Figure 2C) of the sow gut microbial community. For beta diversity analysis, the distribution of microbiota in sow feces with a diet supplemented with silymarin in late pregnancy and lactation was significantly different from that in the control group (Figure 3A). Cluster analysis was performed on the samples, and a PCoA cluster diagram was constructed. The silymarin treatment group and the control group had apparent clusters according to the principal coordinate analysis (Figure 3B).

### 3.4. Changes in Fecal Microbiota Composition by Silymarin Supplementation in Lactating Sows

**Figure 4:**
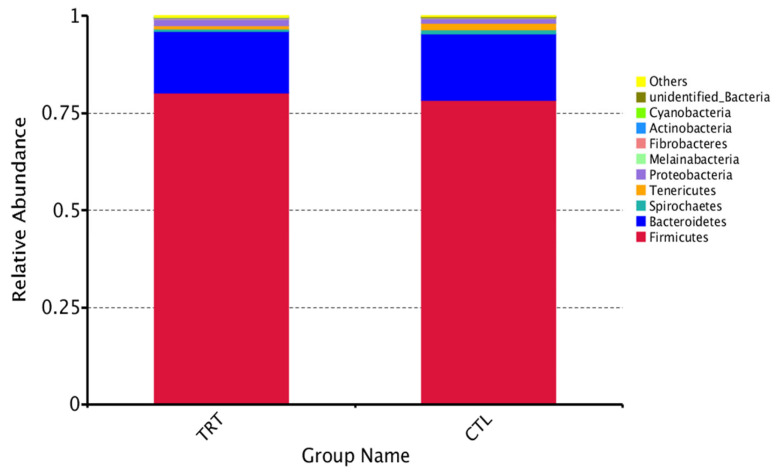
16S rRNA gene analysis revealed phylum-level differences in lactation sow feces between treatments. *n* = 10. CTL, sow fed the basal diet; TRT, sow fed basal diet with silymarin 40 g/d from late gestation to lactation.

**Figure 5:**
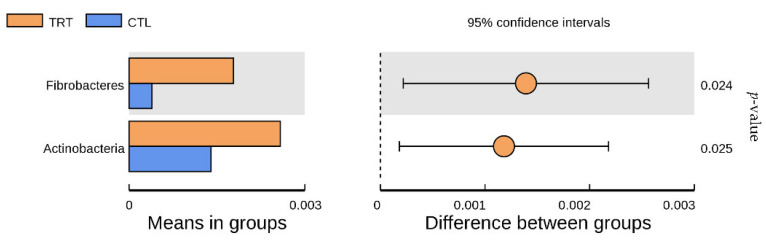
Analysis of distinct species between treatment groups at the phylum level. *n* = 10. CTL, sow fed the basal diet; TRT, sow fed basal diet with silymarin 40 g/d from late gestation to lactation.

When silymarin was supplemented to the diet of sows during late gestation and lactation, there were eight dominant phyla with an average relative abundance > 0.1% in the treatment and control groups: Firmicutes, Bacteroidetes, Spirochaetaes, Tenericutes, Proteobacteria, Melainabacteria, Fibrobacteres, and Actinobacteria (Figure 4). Among them, Firmicutes and Bacteroidetes were the most abundant, accounting for more than 95% of the phyla in the samples. Silymarin significantly increased the relative abundance of Fibrobacteres and Actinobacteria (*p* < 0.05, Figure 5), and tended to reduce the relative abundance of Spirochaetaes and Tenericutes (*p* = 0.09, 0.06, Table 3) at the phylum level.

**Figure 6:**
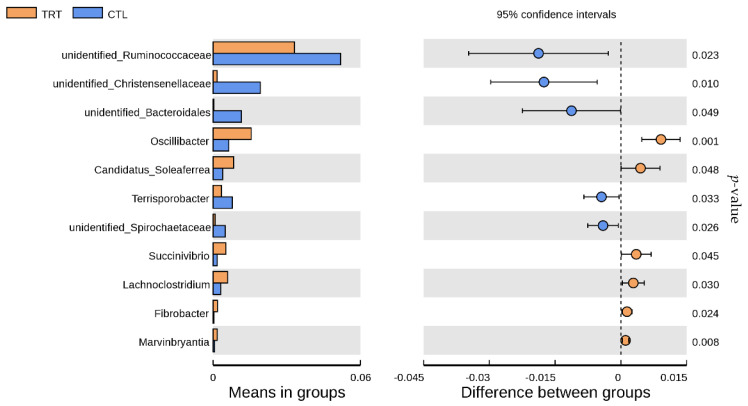
Analysis of distinct species between treatment groups at the genus level. *n* = 10. CTL, sow fed the basal diet; TRT, sow fed basal diet with silymarin 40 g/d from late gestation to lactation.

At the genus level, 11 genera relative abundances changed on the 18th day of lactation based on the dietary silymarin supplementation during late gestation and lactation (Figure 6, Table 4). Silymarin supplementation reduced the relative abundance of *unidentified_Ruminococcaceae*, *unidentified_Bacteroidales*, *Terrisporobacter,* and *unidentified_Spirochaetaceae* (*p* < 0.05), and increased the relative abundance of *Oscillibacter*, *Candidatus_Soleaferrea*, *Succinivibrio*, *Lachnoclostridium*, *Fibrobacter*, and *Marvinbryantia* at the genus level (*p* < 0.05).

### 3.5. Relationship between Gut Microbiota and Blood Cytokines in Lactating Sows

**Figure 7:**
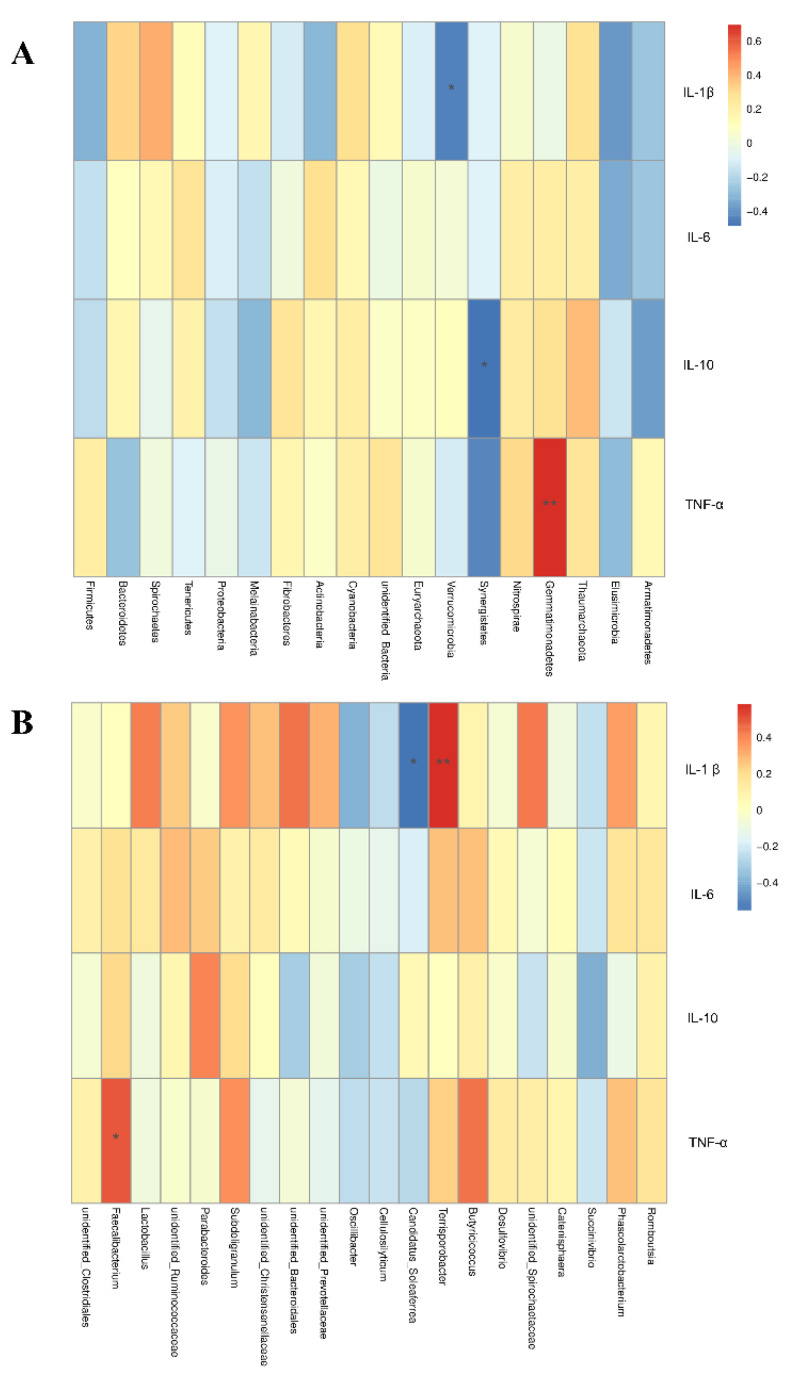
Heat map of the Spearman r correlations between gut microbiota and plasma cytokines in lactation sow at the phylum level (**A**) and the genus level (**B**). *n* = 10. * is significantly different (*p* < 0.05). ** are significantly different (*p* < 0.01) (following the Spearman correlation analysis).

Spearman correlation analysis was used to study the relationship between serum cytokines and microbial species richness. At the phylum level, Gemmatimonadetes was positively correlated with plasma TNF-α (r = 0.69, *p* < 0.01; Figure 7A). Spirochaetes tended to be positively correlated with serum IL-1β (r = 0.41, *p* = 0.07). Verrucomicrobia was negatively correlated with serum IL-1β (r = −0.45, *p* < 0.05). Synergistetes was negatively correlated with serum IL-10 (r = −0.49, *p* < 0.05). At the genus level, *Terrisporobacter* was positively correlated with serum IL-1β (r = 0.58, *p* < 0.01; Figure 7B). However, *Candidatus_Soleaferrea* was negatively correlated with serum IL-1β (r = −0.55, *p* < 0.05). *Faecalibacterium* was positively correlated with serum TNF-α (r = 0.50, *p* < 0.05).

## 4. Discussion

In late gestation and lactation, sows face stresses, such as changing stalls, changes in diet, and parturition. These factors change the intestinal microflora, reduce the disease resistance of sow, and are prone to bacterial infection and cause mastitis, which will seriously affect milk production [16]. In this study, although no significant effect on inflammatory factors was found in milk, serum IL-lβ was significantly reduced on the 18th day of lactation after dietary silymarin supplementation, and there were no significant changes in TNF-α, IL-6, and IL-10. However, our previous study found a decrease in TNF-α concentration in serum on the seventh day of lactation. This agrees with Giorgi et al. [17], who found that silibinin can reduce the levels of NF-κB and cytokines TNF-α and IL-1β in preeclamptic women. Studies have shown that silymarin can inhibit the expression of TNF-α [3,18], *IL-2*, *IL-4*, and *IFN-γ* mRNA expression and, at the same time, increase the expression of *IL-10* [19], thereby inhibiting the occurrence of inflammation. The dynamics of pro- and anti-inflammatory cytokines changes in sow serum during the peripartum period (day −28 to +28) evaluation found that the levels of IL-8 and IL-10 were stable in healthy sows [20]. No significant changes were observed in IL-10. This result may reflect the fact that the sows in the present experiment were in a healthy state. Interestingly, we also found a downward trend in the log10 somatic cell count in colostrum in this study, one of the best indicators of mastitis status [21]. It has been found in other studies that animals with lower somatic cell counts have higher milk production [21,22,23]. Therefore, this could reflect that silymarin can reduce the sow inflammatory response, improve mammary gland health, and thus improve lactation performance. This was consistent with our previously reported findings that silymarin supplementation in the sow before farrowing and lactation increased sow colostrum production and significantly increased average piglet weaning weight and daily gain [3].

This study was the first to focus on the effect of dietary silymarin supplementation from late gestation to lactation on gut microbial composition in lactation sow. Standardized silymarin extracts contain approximately 65–80% flavonolignans, which were obtained from *S. marianum* seeds with small amounts of flavonoids [24]. Flavonolignans have an inhibitory effect on intestinal flora metabolism at a concentration of 200 mg/L [25]. It was observed in this study that the supplementation of silymarin in the sow diet in late gestation and lactation reduced the number of OUTs and community richness indices (observed_species, ACE, Chao1) of intestinal flora and changed the structure of intestinal flora. An experiment in mice revealed that silibinin modulates the abundance of several critical bacterial groups involved in the development of Alzheimer’s disease and tends to reduce the diversity of bacterial flora [26]. The gut microbial diversity of sow decreased significantly after receiving 1.0 kg/t lysozyme for 21 days before farrowing (the study started 24 days before the expected farrowing date) [27]. Reduced microbial diversity may indicate a better intestinal condition and physiological preparation for parturition [14,28]. However, a formula diet alters the colon microbiota and appears to shift the tryptophan metabolism from serotonin to tryptamine, leading to greater histamine levels and the risk of allergies in infants [29]. It was found in this study that dietary silymarin supplementation reduced lactation sow gut microbiota diversity. Further studies are needed to elucidate the impact of this reduction in microbial diversity on sows or offspring.

Abundant Firmicutes and Bacteroidetes were found in the feces of the sows evaluated in this study. The abundance of the two groups accounted for more than 95%. The ratio of Firmicutes and Bacteroidetes is related to the energy metabolism of the body [30]. The difference in the ratio was insignificant in this study, indicating that silymarin supplementation did not change the ratio of Firmicutes and Bacteroidetes and further affected intestinal energy metabolism in sows. However, silymarin treatment tended to reduce the relative abundance of Spirochaetaes and Tenericutes at the phylum level and reduced genera abundance, including *unidentified_Ruminococcaceae*, *Terrisporobacter*, *unidentified_Christensenellaceae*, *unidentified_Bacteroidales,* and *unidentified_Spirochaetaceae*, which reduced the relative abundance of some potential pathogenic bacteria [31,32,33]. Similar to the results of this study, our previous study found that lysozyme supplementation in sow diets reduced the concentration of inflammatory factors and, at the same time, decreased the abundance of Spirochaetaes and Tenericutes at the phylum level in sow gut microbiota [13]. In this study, Spearman correlation analysis found that Spirochaetaes had a positive correlation trend with IL-1β. This finding was confirmed with dietary supplementation with silymarin-reduced serum IL-1β concentrations. It has been found in previous studies that silymarin has inhibitory effects on both gram-positive and gram-negative bacteria. Additionally, silymarin has a more substantial inhibitory effect on gram-positive bacteria, where a minimum inhibitory concentration of 12.5–1000 μg/mL also inhibited the formation of biofilms [34,35]. No significant differences were observed between Gram-negative and Gram-positive bacteria in this study. Following silymarin supplementation, it was found that the relative abundance of phyla Fibrobacteres, Actinobacteria, and genera *Oscillibacter*, *Candidatus_Soleaferrea*, *Succinivibrio*, *Lachnoclostridium*, *Fibrobacter,* and *Marvinbryantia* increased significantly. These bacteria may be associated with the metabolism of anti-inflammatory products and cellulolysis [36,37,38]. These results suggest that silymarin supplementation can reduce inflammatory responses while reducing harmful microbes and increasing the abundance of beneficial microbes.

## 5. Conclusions

In conclusion, in this study, we suggested that dietary silymarin supplementation from late gestation to lactation altered gut microbiota composition, reducing inflammatory responses. This was indicated by the decreased blood concentration of IL-1β and a tendency to decrease colostrum somatic cell count in lactating sows. There has been little knowledge about the lactation sow microbial alterations induced by silymarin supplementation, meaning that more attention should be given to future research. Further studies are needed to elucidate the mechanisms underlying the interaction between gut microbiota and inflammatory responses in sows following dietary silymarin supplementation.

## Figures and Tables

**Table 1:** Effects of dietary silymarin supplementation during late gestation and lactation on the somatic cell count in sow milk.

	CTL	TRT	*p*-Value
Colostrum (log10/mL)	5.83 ± 0.10	5.57 ± 0.08	0.06
Milk (log10/mL)	5.69 ± 0.11	5.51 ± 0.07	0.17

Data are expressed as means ± standard error, *n* = 10 for each group (parity 2 to 4). CTL, sow fed the basal diet; TRT, sow fed basal diet with silymarin 40 g/d from late gestation to lactation.

**Table 2:** Effects of dietary silymarin supplementation during late gestation and lactation on the cytokines in blood and milk serum of lactating sows.

	CTL	TRT	*p*-Value
Blood			
IL-1β, ng/mL	0.10 ± 0.02 ^a^	0.04 ± 0.01 ^b^	<0.01
IL-6, ng/mL	1.13 ± 0.26	0.78 ± 0.18	0.48
IL-10, ng/mL	0.69 ± 0.13	0.72 ± 0.13	0.89
TNF-α, ng/mL	0.30 ± 0.06	0.23 ± 0.04	0.59
Milk serum			
IL-1β, pg/L	73.66 ± 6.41	85.23 ± 8.69	0.30
IL-6, pg/mL	84.71 ± 11.22	75.10 ± 12.63	0.58
IL-10, pg/mL	17.43 ± 3.09	12.71 ± 1.95	0.23
TNF-α, pg/mL	25.57 ± 2.08	28.04 ± 2.36	0.44

Data are expressed as means ± standard error, *n* = 10 for each group (parity 2 to 4). CTL, sow fed the basal diet; TRT, sow fed basal diet with silymarin 40 g/d from late gestation to lactation. ^a,b^ Means not sharing identical superscripts in the same row are significantly different (*p* < 0.05).

**Table 3:** The effect of dietary silymarin supplementation on the relative abundances of eight phyla (%, the relative abundance > 0.1%) in sow feces and Firmicutes/Bacteroidetes ratio.

Item	CTL	TRT	*p*-Value
Firmicutes	78.25 ± 6.99	80.23 ± 5.54	0.49
Bacteroidetes	17.19 ± 6.07	15.94 ± 5.24	0.63
Spirochaetes	1.16 ± 0.96	0.51 ± 0.62	0.09
Tenericutes	1.53 ± 0.86	0.77 ± 0.79	0.06
Proteobacteria	1.28 ± 0.32	1.79 ± 0.82	0.10
Melainabacteria	0.12 ± 0.21	0.01 ± 0.01	0.12
Fibrobacteres	0.04 ± 0.06 ^b^	0.18 ± 0.16 ^a^	0.02
Actinobacteria	0.14 ± 0.04 ^b^	0.26 ± 0.14 ^a^	0.02
Firmicutes/Bacteroidetes	5.77 ± 2.19	5.26 ± 2.43	0.93

Data are expressed as mean ± SD. *n* = 10. CTL, sow fed the basal diet; TRT, sow fed basal diet with silymarin 40 g/d from late gestation to lactation. ^a,b^ Means not sharing identical superscripts in the same row are significantly different (*p* < 0.05).

**Table 4:** The effect of dietary silymarin supplementation on the relative abundances at the genus level (%, the relative abundance > 0.15%) in lactation sow feces.

Item	CTL	TRT	*p*-Value
*unidentified_Clostridiales*	7.11 ± 4.46	8.65 ± 5.28	0.83
*Faecalibacterium*	2.29 ± 1.63	0.72 ± 0.93	0.91
*Lactobacillus*	4.49 ± 2.96	2.09 ± 2.09	0.93
*unidentified_Ruminococcaceae*	5.19 ± 1.70 ^a^	3.32 ± 1.69 ^b^	0.02
*Parabacteroides*	1.10 ± 0.54	2.29 ± 1.83	0.18
*Subdoligranulum*	0.85 ± 1.61	0.09 ± 0.12	0.31
*unidentified_Christensenellaceae*	1.92 ± 1.69 ^a^	0.17 ± 0.13 ^b^	0.01
*unidentified_Bacteroidales*	1.16 ± 1.57	0.03 ± 0.03	<0.05
*unidentified_Prevotellaceae*	1.48 ± 1.35	0.57 ± 0.84	0.20
*Oscillibacter*	0.64 ± 0.27 ^b^	1.55 ± 0.58 ^a^	<0.01
*Cellulosilyticum*	0.20 ± 0.26	0.38 ± 0.11	0.27
*Candidatus_Soleaferrea*	0.39 ± 0.23	0.84 ± 0.60	<0.05
*Terrisporobacter*	0.78 ± 0.51 ^a^	0.34 ± 0.22 ^b^	0.03
*Butyricicoccus*	0.65 ± 0.35	0.56 ± 0.51	0.90
*Desulfovibrio*	0.88 ± 0.27	0.81 ± 0.47	0.93
*unidentified_Spirochaetaceae*	0.49 ± 0.48 ^a^	0.08 ± 0.08 ^b^	0.03
*Catenisphaera*	0.18 ± 0.12	0.43 ± 0.39	0.19
*Succinivibrio*	0.17 ± 0.10	0.52 ± 0.47	0.04
*Phascolarctobacterium*	0.75 ± 0.34	0.44 ± 0.28	0.06
*Romboutsia*	0.37 ± 0.34	0.36 ± 0.21	0.99
*Lachnoclostridium*	0.31 ± 0.19 ^b^	0.59 ± 0.32 ^a^	0.03
*Alloprevotella*	0.39 ± 0.36	0.18 ± 0.12	0.09
*Blautia*	0.13 ± 0.12	0.32 ± 0.28	0.17
*Fibrobacter*	0.04 ± 0.07 ^b^	0.18 ± 0.16 ^a^	0.02
*Marvinbryantia*	0.06 ± 0.05 ^b^	0.17 ± 0.10 ^a^	<0.01

Data are expressed as mean ± SD. *n* = 10. CTL, sow fed the basal diet; TRT, sow fed basal diet with silymarin 40 g/d from late gestation to lactation. ^a,b^ Means not sharing identical superscripts in the same row are significantly different (*p* < 0.05).

## Data Availability

The datasets presented in this study can be found online repositories. The names of the repository/repositories and accession number can be found at: https://www.ncbi.nlm.nih.gov/, BioProject ID PRJNA842556. Further inquiries can be directed to the corresponding author.
